# Supplementary material for: Prevalence, awareness, treatment, and control of hypertension in Bangladesh: Findings from National Demographic and Health Survey, 2017–2018
Source: J Clin Hypertens (Greenwich). 2021 Sep 7;23(10):1830–42. doi: 10.1111/jch.14363 (PMC8678656; doi:10.1111/jch.14363)
Supplement: Supplementary file 1 — Supporting Information [file JCH-23-1830-s001.docx]

**Supplementary table 1: Factors associated with hypertension, awareness, treatment and control in adults aged ≥18 years in the Bangladeshi population, BDHS 2017-18**

| **Characteristics** | **Hypertension, PR (95%CI)** | | | **Awarness, PR (95%CI)** | | | **Treatment, PR (95%CI)** | | | **Control, PR (95%CI)** | | |
| --- | --- | --- | --- | --- | --- | --- | --- | --- | --- | --- | --- | --- |
|  | **Model 1** | **Model 2** | **Model 3** | **Model 1** | **Model 2** | **Model 3** | **Model 1** | **Model 2** | **Model 3** | **Model 1** | **Model 2** | **Model 3** |
| **Individual level** |  |  |  |  |  |  |  |  |  |  |  |  |
| ***Age in years (ref: 18-34)*** |  |  |  |  |  |  |  |  |  |  |  |  |
| 35-39 | 2.09 (1.85-2.37)^***^ | 2.10 (1.85-2.37)^***^ | 2.08 (1.84-2.35)^***^ | 1.12 (0.90-1.37) | 1.10 (0.89-1.36) | 1.10 (0.89-1.36) | 1.28 (1.01-1.63)^***^ | 1.26 (1.00-1.61)^**^ | 1.26 (0.99-1.59) | 0.83 (0.62-1.13) | 0.84 (0.62-1.14) | 0.82 (0.61-1.11) |
| 40-44 | 2.38 (2.09-2.71)^***^ | 2.38 (2.09-2.71)^***^ | 2.34 (2.06-2.66)^***^ | 1.53 (1.28-1.82)^***^ | 1.52 (1.28-1.80)^***^ | 1.51 (1.27-1.80)^***^ | 1.70 (1.38-2.10)^***^ | 1.69 (1.37-2.08)^***^ | 1.68 (1.13-1.55)^***^ | 0.74 (0.55-1.00)^*^ | 0.74 (0.55-1.01) | 0.73 (0.55-0.98)^*^ |
| 45-49 | 2.89 (2.56-3.26)^***^ | 2.88 (2.55-3.25)^***^ | 2.83 (2.51-3.20)^***^ | 1.57 (1.32-1.87)^***^ | 1.52 (1.28-1.81)^***^ | 1.52 (1.28-1.81)^***^ | 1.92 (1.57-2.36)^***^ | 1.85 (1.51-2.27)^***^ | 1.87 (1.52-2.29)^***^ | 0.62 (0.46-0.83)^***^ | 0.62 (0.46-0.84)^***^ | 0.64 (0.48-0.86)^***^ |
| 50-54 | 3.39 (2.98-3.86)^***^ | 3.39 (2.98-3.85)^***^ | 3.32 (2.95-3.78)^***^ | 1.77 (1.49-2.11)^***^ | 1.72 (1.44-2.05)^***^ | 1.72 (1.45-2.05)^***^ | 2.00 (1.62-1.47)^***^ | 1.93 (1.56-2.38)^***^ | 1.94 (1.58-2.40)^***^ | 0.69 (0.50-0.94)^***^ | 0.69 (0.50-0.94)^***^ | 0.70 (0.51-0.96)^*^ |
| 55-59 | 3.76 (3.35-4.23)^***^ | 3.76 (3.34-4.23)^***^ | 3.65 (3.24-4.11)^***^ | 2.01 (1.70-2.38)^***^ | 1.96 (1.65-2.32)^***^ | 1.96 (1.66-2.33)^***^ | 2.39 (1.98-2.90)^***^ | 2.32 (1.91-2.82)^***^ | 2.34 (1.93-2.85)^***^ | 0.69 (0.51-0.92)^***^ | 0.70 (0.52-0.93)^*^ | 0.72 (0.53-0.96)^*^ |
| 60-64 | 4.26 (3.78-4.80)^***^ | 4.25 (3.77-4.79)^***^ | 4.10 (3.64-4.63)^***^ | 2.14 (1.82-2.51)^***^ | 2.09 (1.78-2.46)^***^ | 2.10 (1.79-2.47)^***^ | 2.60 (2.15-3.14)^***^ | 2.54 (2.10-3.06)^***^ | 2.57 2.13-3.10)^***^ | 0.67 (0.49-0.92)^***^ | 0.68 (0.50-0.93)^*^ | 0.70 (0.51-0.95)^*^ |
| ≥65 | 5.15 (4.60-5.76)^***^ | 5.14 (4.59-5.75)^***^ | 4.99 (4.46-5.59)^***^ | 2.07 (1.76-2.45)^***^ | 2.02 (1.71-2.39)^***^ | 2.03 (1.72-2.40)^***^ | 2.46 (2.02-2.99)^***^ | 2.38 (1.96-2.89)^***^ | 2.41 (1.98-2.92)^***^ | 0.45 (0.33-0.61)^***^ | 0.45 (0.33-0.62)^***^ | 0.47 (0.35-0.64)^***^ |
| ***Sex (ref: men)*** | 1.16 (1.08-1.25)^***^ | 1.16 (1.08-1.25)^***^ | 1.15 (1.07-1.23) ^***^ | 1.49 (1.34-1.66)^***^ | 1.52 (1.36-1.69)^***^ | 1.53 (1.37-1.70)^***^ | 1.52 (1.35-1.71)^***^ | 1.55 (1.37-1.74)^***^ | 1.57 (1.39-1.79)^***^ | 1.05 (0.84-1.31) | 1.05 (0.84-1.31) | 1.08 (0.87-1.36) |
| ***Body Mass Index (kg/m^2^) (ref: normal weight)*** |  |  |  |  |  |  |  |  |  |  |  |  |
| Underweight (<18.5) | 0.71 (0.64-0.79)^***^ | 0.72 (0.65-0.79)^***^ | 0.73 (0.66-0.80)^***^ | 0.77 (0.66-0.91)^***^ | 0.81 (0.68-0.95)^***^ | 0.80 (0.67-0.94)^***^ | 0.75 (0.62-0.91)^***^ | 0.79 (0.65-0.95)^***^ | 0.77 (0.64-0.93)^***^ | 0.98 (0.70-1.36) | 0.99 (0.71-1.37) | 0.95 (0.69-1.31) |
| Overweight (23.0-27.5) | 1.58 (1.47-1.70)^***^ | 1.58 (1.47-1.70)^***^ | 1.58 (1.47-1.70)^***^ | 1.20 (1.09-1.33)^***^ | 1.17 (1.06-1.30)^***^ | 1.17 (1.06-1.30)^***^ | 1.27 (1.13-1.42)^***^ | 1.23 (1.10-1.38)^*^ | 1.22 (1.09-1.37)^***^ | 0.77 (0.63-0.95)^***^ | 0.78 (0.63-0.96)^*^ | 0.78 (0.64-0.96)^*^ |
| Obesity (>27.5) | 1.87 (1.72-2.04)^***^ | 1.86 (1.71-2.04)^***^ | 1.87 (1.71-2.04)^***^ | 1.34 (1.19-1.51)^***^ | 1.26 (1.12-1.43)^***^ | 1.27 (1.12-1.43)^***^ | 1.36 (1.19-1.56)^***^ | 1.28 (1.11-1.47)^***^ | 1.28 (1.11-1.47)^***^ | 0.97 (0.77-1.21) | 0.98 (0.78-1.23) | 0.98 (0.78-1.25) |
| **Respondents education (ref: higher education)** |  |  |  |  |  |  |  |  |  |  |  |  |
| No education/pre-primary | 0.91 (0.82-1.02) | 0.92 (0.82-1.03) | 0.96 (0.86-1.08) | 0.82 (0.70-0.96)^*^ | 0.92 (0.78-1.08) | 0.91 (0.77-1.07) | 0.87 (0.73-1.04) | 0.98 (0.82-1.18) | 10.96 (0.80-1.16) | 0.70 (0.52-0.95)^*^ | 0.68 (0.50-0.93)^*^ | 0.70 (0.52-0.95)^*^ |
| Primary | 0.93 (0.84-1.04) | 0.94 (0.84-1.05) | 0.96 (0.86-1.07) | 0.96 (0.83-1.11) | 1.05 (0.90-1.21) | 1.03 (0.89-1.20) | 0.99 (0.84-1.16) | 1.07 (0.91-1.27) | 1.04 (0.88-1.23) | 0.85 (0.65-1.11) | 0.83 (0.63-1.11) | 0.81 (0.61-1.06) |
| Secondary | 0.97 (0.88-1.08) | 0.98 (0.88-1.08) | 0.99 (0.90-1.10) | 0.99 (0.86-1.14) | 1.03 (0.89-1.18) | 1.01 (0.88-1.17) | 1.05 (0.90-1.24) | 1.09 (0.92-1.28) | 1.07 (0.91-1.26) | 0.86 (0.67-1.11) | 0.84 (0.65-1.09) | 0.83 (0.65-1.07) |
| ***Currently working (ref: no)*** | 0.96 (0.90-1.04) | 0.97 (0.90-1.04) | 0.96 (0.89-1.03) | *0.93 (0.84-1.03)* | *0.97 (0.88-1.18)* | *0.99 (0.89-1.10)* | 0.92 (0.82-1.02) | 0.95 (0.85-1.07) | *1.00 (0.88-1.12)* | *0.98 (0.80-1.20)* | *0.97 (0.79-1.19)* | *1.07 (0.88-1.31)* |
| ***Diabetes (ref: no)*** | 1.17 (1.09-1.25)^***^ | 1.17 (1.09-1.25)^***^ | 1.19 (1.11-1.28)^***^ | 1.22 (1.12-1.33)^***^ | 1.18 (1.08-1.29)^***^ | 1.17 (1.07-1.28)^***^ | 1.27 (1.15-1.40)^***^ | 1.23 (1.12-1.35)^***^ | 1.21 (1.10-1.33)^***^ | 0.89 (0.73-1.08) | 0.90 (0.74-1.11) | 0.92 (0.75-1.12) |
| **Household level** |  |  |  |  |  |  |  |  |  |  |  |  |
| ***Wealth quintile (ref: lowest)*** |  |  |  |  |  |  |  |  |  |  |  |  |
| Second |  | 1.03 (0.92-1.15) | 1.04 (0.93-1.17) |  | 1.16 (0.97-1.38) | 1.15 (0.96-1.37) |  | 1.15 (0.95-1.38) | 1.13 (0.93-1.36) |  | 1.02 (0.72-1.44) | 1.00 (0.71-1.40) |
| Middle |  | 1.01 (0.91-1.12) | 1.04 (0.93-1.17) |  | 1.26 (1.07-1.49)^***^ | 1.24 (1.05-1.47)^***^ |  | 1.34 (1.13-1.59)^***^ | 1.30 (1.10-1.54)^***^ |  | 0.95 (0.66-1.37) | 0.95 (0.66-1.37) |
| Fourth |  | 1.03 (0.92-1.15) | 1.09 (0.97-1.22) |  | 1.41 (1.20-1.67)^***^ | 1.37 (1.15-1.62)^***^ |  | 1.45 (1.22-1.72)^***^ | 1.37 (1.15-1.63)^***^ |  | 1.10 (0.79-1.53) | 1.06 (0.76-1.49) |
| Highest |  | 1.03 (0.92-1.14) | 1.11 (0.99-1.25) |  | 1.39 (1.18-1.64)^***^ | 1.33 (1.11-1.58)^***^ |  | 1.45 1.21-1.72)^***^ | 1.32 (1.10-1.59)^***^ |  | 0.94 (0.67-1.32) | 0.88 (0.60-1.27) |
| **Community level** |  |  |  |  |  |  |  |  |  |  |  |  |
| ***Place of residence (ref: rural)*** |  |  | 1.02 (0.95-1.10) |  |  | 1.04 (0.95-1.15) |  |  | 1.07 (0.97-1.18) |  |  | 1.07 (0.88-1.30) |
| ***Administrative division (ref: Barishal)*** |  |  |  |  |  |  |  |  |  |  |  |  |
| Chattogram |  |  | 0.92 (0.81-1.04) |  |  | 0.97 (0.82-1.15) |  |  | 1.02 (0.87-1.20) |  |  | 1.22 (0.88-1.69) |
| Dhaka |  |  | 0.75 (0.66-0.86)^***^ |  |  | 0.98 (0.82-1.15) |  |  | 0.99 (0.84-1.18) |  |  | 0.94 (0.65-1.35) |
| Khulna |  |  | 0.90 (0.80-1.01) |  |  | 0.96 (0.81-1.14) |  |  | 0.91 (0.76-1.09) |  |  | 0.75 (0.52-1.07) |
| Mymensingh |  |  | 0.80 (0.70-0.91)^***^ |  |  | 0.99 (0.83-1.20) |  |  | 1.05 (0.88-1.26) |  |  | 1.39 (0.99-1.95) |
| Rajshahi |  |  | 0.95 (0.83-1.08) |  |  | 0.95 (0.79-1.13) |  |  | 0.84 (0.69-1.02) |  |  | 0.67 (0.44-1.02) |
| Rangpur |  |  | 1.08 (0.96-1.21) |  |  | 0.84 (0.69-1.01) |  |  | 0.76 (0.62-0.93)^***^ |  |  | 0.81 (0.55-1.19) |
| Sylhet |  |  | 0.94 (0.83-1.08) |  |  | 1.13 (0.94-1.36) |  |  | 1.22 (1.02-1.47)^***^ |  |  | 1.10 (0.78-1.55) |

*p value <0.05

***p value <0.01

Hypertension is defined as having systolic blood pressure ≥140 mmHg and/or a diastolic blood pressure ≥90 mmHg, or taking any prescribed drugs to control blood pressure. Awareness of hypertension was defined as a self-reported previous diagnosis of hypertension by a doctor or nurse in people with confirmed hypertension. Treatment of hypertension was defined as self-reported use of a prescription antihypertensive medication for management of hypertension. Control of hypertension was defined as receiving antihypertensive medication and having an average systolic blood pressure below 140mmHg and/or diastolic blood pressure below 90 mmHg
